# The Role of Dislocation Type in the Thermal Stability of Cellular Structures in Additively Manufactured Austenitic Stainless Steel

**DOI:** 10.1002/advs.202402962

**Published:** 2024-07-01

**Authors:** Dayong An, Yao Xiao, Junshi Yu, Xu Zhang, Zan Li, Yan Ma, Rui Li, Xianhong Han, Xifeng Li, Jun Chen, Stefan Zaefferer

**Affiliations:** ^1^ Department of Plasticity Technology School of Materials Science and Engineering Shanghai Jiao Tong University Shanghai 200030 P. R. China; ^2^ Institute of Clean Energy Yangtze River Delta Research Institute Northwestern Polytechnical University Taicang 215400 P. R. China; ^3^ Applied Mechanics and Structure Safety Laboratory of Sichuan Province School of Mechanics and Aerospace Engineering Southwest Jiaotong University Chengdu 610031 P. R. China; ^4^ State Key Laboratory of Metal Matrix Composites Shanghai Jiao Tong University Shanghai 200240 P. R. China; ^5^ Max‐Planck‐Institut für Eisenforschung 40237 Düsseldorf Germany

**Keywords:** cellular structure, dislocation type, in situ observation, laser powder‐bed fusion, thermal stability

## Abstract

The ultrafine cellular structure promotes the extraordinary mechanical performance of metals manufactured by laser powder‐bed‐fusion (L‐PBF). An in‐depth understanding of the mechanisms governing the thermal stability of such structures is crucial for designing reliable L‐PBF components for high‐temperature applications. Here, characterizations and 3D discrete dislocation dynamics simulations are performed to comprehensively understand the evolution of cellular structures in 316L stainless steel during annealing. The dominance of screw‐type dislocation dipoles in the dislocation cells is reported. However, the majority of dislocations in sub‐grain boundaries (SGBs) are geometrically necessary dislocations (GNDs) with varying types. The disparity in dislocation types can be attributed to the variation in local stacking fault energy (SFE) arising from chemical heterogeneity. The presence of screw‐type dislocations facilitates the unpinning of dislocations from dislocation cells/SGBs, resulting in a high dislocation mobility. In contrast, the migration of SGBs with dominating edge‐type GNDs requires collaborative motion of dislocations, leading to a sluggish migration rate and an enhanced thermal stability. This work emphasizes the significant role of dislocation type in the thermal stability of cellular structures. Furthermore, it sheds light on how to locally tune dislocation structures with desired dislocation types by adjusting local chemistry‐dependent SFE and heat treatment.

## Graphical abstract

This study explores the mechanisms underlying the variation in dislocation types within cellular structures of additively manufactured (AMed) 316L stainless steel. Results address the fundamental role of dislocation types in their behavior upon annealing. Understanding the formation and evolution of cellular structures is of both fundamentally and technologically important, offering a new strategy for designing microstructures in AMed components.

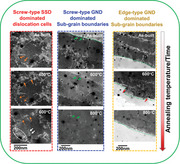

## 1 Introduction

Laser powder‐bed‐fusion (L‐PBF) is one of the most prevalent metal additive manufacturing (AM) technologies, which offers many advantages in producing net‐shaped components with complex geometries, high accuracies, and superior properties.^[^ ^1^ ^]^ Due to the large thermal gradients and rapid cooling rates, sub‐micrometer cellular structures are widely present in many L‐PBF materials with a face‐centered cubic (FCC) crystal structure, e.g., austenitic stainless steel,^[^ ^2^ ^]^ nickel‐based superalloy,^[^ ^3^ ^]^ and high‐entropy alloys.^[^ ^4^ ^]^ The sub‐micrometer cellular structures typically possess a high density of dislocations in the cell walls (≈10^14^–10^15^ m^−2^),^[^ ^5^ ^]^ chemical inhomogeneity, and nano‐precipitates.^[^ ^6^ ^]^ Such microstructures render superior mechanical performance in AM parts.^[^ ^7^ ^]^

AM components usually experience elevated temperatures in practical applications or post‐heat treatment. The latter is often needed to alleviate the high residual stress built up during L‐PBF.^[^ ^8^ ^]^ Several studies revealed that the thermal stability of L‐PBF components is significantly different from that of their conventional counterparts.^[^ ^9^ ^]^ At elevated temperatures, the microstructural features of cellular structures, e.g., dislocation cells, may get recovered, leading to a degrading of mechanical properties.^[^ ^10^ ^]^ It was found that the dislocation cells remain stable upon annealing treatment up to 600 °C,^[^ ^10^, ^11^ ^]^ while recovery of such structures is readily activated between 600 °C and 1000 °C.^[^ ^12^ ^]^ The low‐angle grain boundaries (LAGBs) display disparate thermal stabilities, i.e., some of them get annihilated at relatively low temperatures, while others remain stable even at 1000 °C.^[^ ^13^ ^]^ However, the underlying mechanisms governing the distinct thermal stabilities remain unclear, even though such fundamental understanding plays a decisive role in designing reliable and advanced L‐PBF components. Nevertheless, the intricate nature of hierarchical microstructural structures, coupled with a lack of near in situ experimental characterizations and challenges in quantifying the crystallographic details of dislocation structures, have thus far impeded our ability to disentangle the specific mechanisms responsible for the thermal stability of the cellular structures.

In this study, quasi in situ electron channeling contrast imaging (ECCI) in conjunction with high‐angular resolution electron backscatter diffraction (HR‐EBSD) was employed to characterize the evolution of dislocation structures within cellular boundaries during annealing. The geometrically necessary dislocations (GNDs), i.e., dislocations with net Burgers vectors that are not null at a given step size, can be probed using HR‐EBSD. All other dislocation arrangements are called statistically stored dislocations (SSDs). While ECCI is capable of detecting the sum of both SSDs and GNDs, it lacks the ability to distinguish between them. The combination of HR‐EBSD and ECCI facilitates a quantitative assessment of the crystallographic details of defect structures, e.g., misorientation of grain boundaries, dislocation arrangements and types (i.e., screw or edge, positive or negative), etc.^[^ ^14^ ^]^ 3D discrete dislocation dynamics (3D‐DDD) simulations were performed to explore the factors determining the dislocation types and thermal stability of cellular structures. These results reveal the complex dynamic behavior of different dislocation structures associated with cellular boundaries, suggesting a crucial role of dislocation types in their thermal stabilities.

## 2 Results and Discussion

### 2.1 Microstructure of As‐Built 316L Stainless Steel

**Figure 1:**
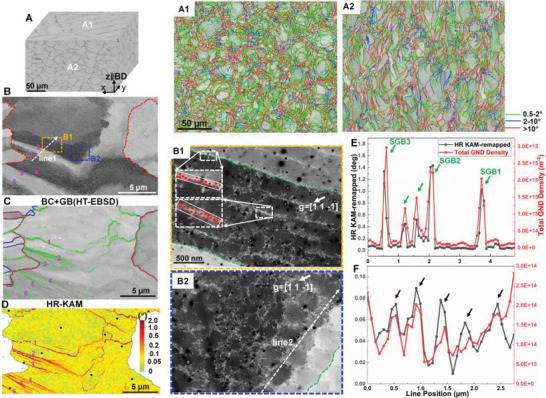
Typical microstructure of a laser powder‐bed‐fusion (L‐PBF) manufactured 316L stainless steel. A) 3D reconstruction of optical microscope (OM) images showing the morphology of melt pool boundaries of the as‐built sample. A1,A2) EBSD‐band contrast (BC) images superimposed with grain boundaries (GBs): A1) xy plane (┴ BD) and A2) xz plane (∥BD). BD: building direction. B) Electron channeling contrast (ECC) image, the sub‐grain boundaries (SGBs) of interest are numbered 1 to 6. B1,B2) The corresponding enlarged ECC images of the regions shown in (B). The dashed green lines in (B1,B2) highlight the position of SGBs. C) EBSD‐BC image superimposed with GBs (based on Hough‐transformation (HT)). D) High‐resolution kernel average misorientation (HR‐KAM) of the selected grain. The black dots in (D) are the selected reference points for cross‐correlation processing. E,F) Profiles of the HR‐KAM values and total GND density along L1 in (B) and L2 in (B2), respectively. The positions of SGBs and dislocation cells are highlighted by green and black arrows in (E) and (F), respectively.

**Figure** **1** shows the microstructure overview of the as‐built 316L stainless steel. The melt pool boundaries are visible in optical microscope images after etching (Figure 1A). The grains display chessboard and ripple patterns in the surfaces perpendicular to (Figure 1A1) and parallel to (Figure 1A2) the building direction (BD), respectively. For both planes, sub‐grain boundaries (SGBs, 0.5‐2° misorientation) constitute around 60% of the total grain boundaries (GBs), which is much more popular than LAGBs (20‐30%, 2–10° misorientation) and high‐angle GBs (HAGBs) (≈10%, >10° misorientation). Figure 1B shows a typical example of the cellular structures probed by ECCI under controlled diffraction conditions (cECCI).^[^ ^14a^ ^]^ The heterogeneous channeling contrast results from the variation of local orientation within the selected grain (explained in Figure S1, Supporting Information), separated by SGBs, as highlighted by green lines in conventional EBSD measurement (Figure 1C, numbered 1‐6). The disconnection of these green lines indicates that some SGBs possess a misorientation angle even less than 0.5°. HR‐EBSD was further employed to quantify this subtle misorientation of SGBs, as displayed in Figure 1D. The cellular structures reveal lamellar (Figure 1B1) or polygonal (Figure 1B2) morphologies, which can be attributed to their different growth directions relative to the observation plane.^[^ ^15^ ^]^

By comparing the ECCI (Figure 1B) and HR‐EBSD (Figure 1D) maps, it can be seen that the SGBs exactly overlap with the cellular boundaries, e.g., green dashed lines in Figure 1B1,B2. This fact is also found in other regions of the sample (Figure S2, Supporting Information) as well as in the literature.^[^ ^13^ ^]^ The dislocation densities in both SGBs and dislocation cell walls, estimated by counting the number of intersections between dislocations and sample surfaces (marked as red dots) and dividing by the selected areas (represented by white rectangles in Figure 1B1), exhibit similar order of magnitude, i.e., approximately 10^15^ m^−2^. Furthermore, the misorientation angle and GND density along SGBs (line 1 in Figure 1B) and dislocation cells (line 2 in Figure 1B2) were quantified using HR‐EBSD, as shown in Figure 1E,F, respectively. SGBs show misorientation angles of 0.4° to 1.6° (Figure 1E), which are substantially higher than that of dislocation cells (0.05° to 0.1°, Figure 1F). As the total dislocation densities of these two microstructural features are similar, their differences in misorientation, thus, must be mostly due to different dislocation types. As depicted in Figure 1E,F, the GND density of SGBs (≈2‐3 × 10^15^ m^−2^) is around one order of magnitude higher than that of the dislocation cell walls (≈2 × 10^14^ m^−2^). This fact indicates that most dislocations at dislocation cell walls are SSDs, which result in extremely low misorientation across dislocation cell walls (that is 0.05° to 0.1°). Furthermore, nano‐precipitates enriched in Mn, O, and Si, and segregation of Cr are probed in the cellular structures, as shown in Figure S3 (Supporting Information).

### 2.2 Dynamic Evolution of Grain Boundaries upon Annealing

**Figure 2:**
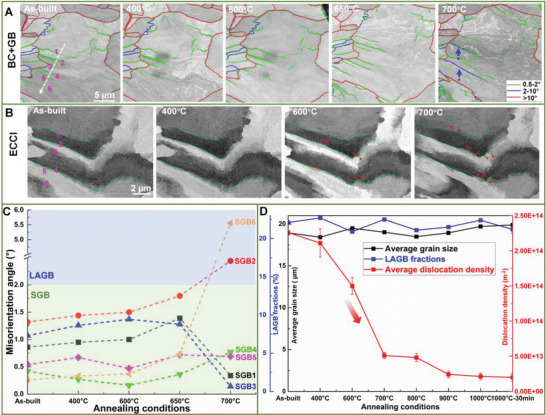
Dynamic evolution of grain boundaries at elevated temperatures. A)Series of EBSD‐BC images superimposed with GBs, and B) series of ECC images of the selected grain. The dashed blue arrows in (A) highlight the newly formed low‐angle GBs (LAGBs), and the red arrows in (B) indicate the migration of sub‐grain boundaries (SGBs). C) Evolution of misorientation angles of SGB1 to SGB6. D) Summaries of the evolution of average grain size, LAGB fractions, and average dislocation density of cellular structures (including the cellular boundaries and interiors). The red arrow in (D) highlights a sharp decrease in dislocation density.

Figure S4 (Supporting Information) displays the dynamic evolution of HAGBs and LAGBs at elevated temperatures to reveal their thermal stability. In general, the fraction of LAGBs (≈25%) and the grain size defined by HAGBs (≈20 µm) remain unchanged at elevated temperatures (summarized in **Figure** **2**D). Since we mainly focus on the cellular structure in this study, the thermal stability of HAGB and LAGB will not be discussed in detail.

Since SGBs constitute the largest fraction of GBs and are coincident with the cellular boundaries, the dynamic evolutions of SGBs and dislocation cells are comprehensively investigated and compared in this study (Figure 2A,B). Based on these quasi in situ EBSD measurements (Figure 2A), the misorientation angle evolutions of SGB1 to SGB6 are summarized in Figure 2C. The change in the misorientation angle of individual SGBs reveals different behaviors upon annealing. For instance, the misorientation angles of SGB1 and SGB3 first increase slightly (by 0.2°−0.5°) up to 650 °C, followed by a substantial decline to nearly 0°. In contrast, the misorientation angles of SGB2 and SGB6 continuously increase and even evolve into LAGBs at 700 °C, as indicated by blue arrows in Figure 2A. Besides, the misorientation angles of SGB4 and SGB5 remain almost constant during annealing up to 700 °C. Apart from the misorientation alterations, the morphologies of SGBs change significantly upon annealing, as demonstrated by a series of ECC images in Figure 2B. Locally, migration of SGBs, indicated by red arrows, is frequently observed when annealing above 600 °C. For example, SGB1 has moved around 1.5 µm after annealing at 700 °C for 10 mins. Interestingly, some of the SGBs become even more curved, e.g., forming humps in the right parts of the green dashed lines (Figure 2B), which could increase the total GB energy. The underlying mechanism for such abnormal morphological change will be discussed in the following section in detail.

### 2.3 Dynamic Evolution of Dislocation Structures upon Annealing

**Figure 3:**
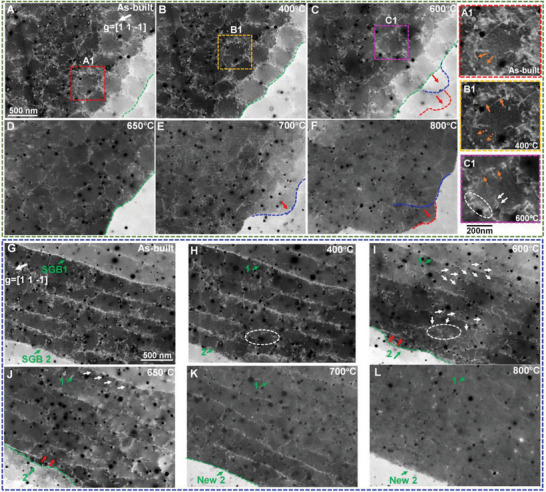
Dynamic evolution of dislocation structures at elevated annealing temperatures probed by ECCI. A–F) Series of ECC images demonstrating the evolution of the region shown in Figure 1B2. A1–C1) Enlarged ECC images of an individual dislocation cell in (A–C). G–L) Series of ECC images demonstrating the evolution of the region shown in Figure 1B1. The dashed green lines in (A–D,I–K) highlight the positions of SGBs. The dashed blue and red lines in (C–F) indicate the pre‐existed cellular boundaries. The red arrows in (C–F,I,J) suggest the migration direction of SGBs. The brown arrows in (A1–C1) demonstrate the annihilation of dislocations within the cellular boundaries. The white arrows in (C1,I,J) depict the process of dislocation unpinning from the cellular boundaries to the channels. The white dashed ellipses in (C1,H,I) reveal the re‐trapping of dislocations by the pre‐existed cellular boundaries.

Significant changes in morphologies and misorientation of SGBs are detected in the annealing temperature ranging from 600 °C to 700 °C (Figure 2A–C)). Meanwhile, a sharp decrease in average dislocation density (from 1.50 ± 0.12 × 10^14^ m^−2^ to 5.11 ± 0.43 × 10^13^ m^−2^, the density estimation process is shown in Figure S5, Supporting Information) is observed during the same annealing range (Figure 2D). This coincidence indicates that the thermal stabilities of both SGBs and dislocation cells are associated with the dislocation annihilation behavior. Therefore, we conducted an in‐depth investigation into the associated dislocation behavior of SGBs and dislocation cells (**Figure** **3**).

In general, dislocation cells reveal lower thermal stability compared with SGBs. When the specimen is annealed up to 400 °C, most of the dislocations remain tangled, while a few of them start to be annihilated (e.g., the regions marked by brown arrows in Figure 3A1,B1). Due to the low mobility of dislocations at this temperature, dislocation annihilation is confined within the original cellular boundaries. Such annihilation behavior is considered as intrinsic dislocation annihilation. With an increase in annealing temperature, dislocation cells start disentangling, and only few elongated dislocations are left at 700 °C (Figure 3C–E). Apart from the intrinsic dislocation annihilation, some dislocations get unpinned from the cell walls and move across the cell interiors (highlighted by white arrows in Figure 3I,J), resulting in dislocation annihilation between neighboring cells. Such annihilation behavior is considered as extrinsic dislocation annihilation. Generally, the intrinsic dislocation annihilation plays a dominant role in the thermal stability of dislocation cells. It is worth mentioning that nano‐precipitates in this material remain stable during annealing up to 1000 °C. The thermally activated dislocations get re‐trapped by their neighboring cellular boundaries decorated with precipitates, as shown in the regions marked by white dashed ellipses in Figure 3C1,H,I.

Regarding the associated dislocation behavior of SGBs, two salient features need to be emphasized from these experimental observations. First, the formed humps along SGBs are always found to be coincident with pre‐existing cellular boundaries, as highlighted by blue and red dashed lines in Figure 3C–F. Migration of SGBs towards pre‐existing cellular boundaries is also observed in Figure 3I–K (as indicated by red arrows). Such movement may be driven by the residual stresses resulting from the L‐PBF process (Figure S6A,B, Supporting Information) or/and the stored dislocation energy. It should be emphasized that, due to the so‐called “reference pattern problem”,^[^ ^16^ ^]^ the absolute value of residual stresses cannot be accurately determined via HR‐EBSD. Nevertheless, the relative stress values still give us an important hint on the driving force for dislocation/SGB migration. Specifically, the alternation of tensile (red color) and compressive (blue color) stresses across SGBs, highlighted by gradient‐colored arrows in Figure S6A,B (Supporting Information), promotes the migration of these boundaries. As the pre‐existing cellular boundary, decorated with high‐density dislocations and/or nanoprecipitates, can act as a strong barrier for dislocation motion,^[^ ^17^ ^]^ the dislocation‐mediated migration of SGBs is, thus, strongly hindered by these cellular boundaries. Secondly, SGB1 and SGB2 show similar GND density before annealing (≈2 × 10^15^ m^−2^, Figure 1E). However, many dislocations get unpinned from SGB1 upon annealing (highlighted by white arrows in Figure 3I,J), deteriorating its thermal stability and enhancing its migration rate. At 800 °C, the SGB1 is hardly recognized and the channeling contrast became homogeneous in the original regions. In contrast, SGB2 shows higher thermal stability and lower mobility, as displayed in Figure 3G–L. Such observations suggest that the absolute value of GND density does not impose a decisive effect on the thermal stability of SGBs.

### 2.4 The Dislocation Types in Different Cellular Structures

**Figure 4:**
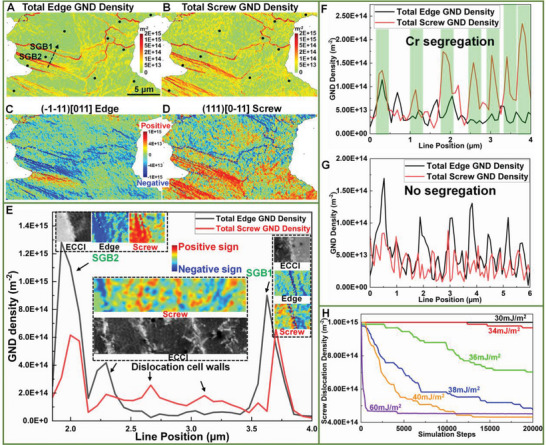
Dislocation types of the cellular structures. A,B) The distribution of A) total edge‐type and B) total screw‐type GNDs. C,D) Distributions of two primary GND types with evident dislocation densities. The red and blue colors indicate the positive and negative signs of dislocations, respectively. E) Profiles of the total edge‐type and total screw‐type GND densities along line1 (within the range between SGB1 and SGB2) in Figure 1B. Insets are the enlarged ECC images and primary GND distributions of the corresponding SGBs and dislocation cells. F,G) Profiles of the total edge‐type and total screw‐type GND densities along F) dislocation structures with Cr‐segregation and G) those without Cr‐segregation. H) 3D discrete dislocation dynamics (3D‐DDD) simulations showing the annihilation rates of screw‐type dislocations as a function of SFE values.

To elucidate the intrinsic reason for the distinct thermal stability of different SGBs and dislocation cells, the dislocation types of these dislocation structures were analyzed using HR‐EBSD. **Figure** **4**A,B presents the distribution of total edge‐type and total screw‐type GNDs, respectively. It is evident that the skeletons of SGBs are mainly composed of edge‐type dislocations, while within sub‐grains (i.e., dislocation cell regions) screw‐type dislocations become more prominent. To ensure the representativity of these findings, we examined the dislocation type distributions in three additional grains exhibiting well‐organized dislocation structures, which show consistent results. Additionally, we calculated the distribution of all GND types (12 edge and 6 screw dislocations). Figure 4C,D illustrates two distinct GND types with evident dislocation densities. Note that, the red and blue colors depict the positive and negative signs of Burgers vectors, respectively. Interestingly, Figure 4D reveals the presence of screw‐type dislocations with opposite signs, specifically within the dislocation cell regions.

To quantitively analyze the dislocation types of dislocation cells and SGBs, profiles of total edge‐type and total screw‐type GNDs along the black dashed arrow (Figure 4A) are plotted in Figure 4E, represented by black and red lines, respectively. Additionally, enlarged ECC images of the corresponding cellular structures (SGBs and dislocation cells), along with the distribution of primary GND types measured by HR‐EBSD, are displayed in the insets of Figure 4E. It can be clearly seen that the screw‐type GNDs with opposite signs (indicated by blue and red color) are arranged on the two sides of dislocation cell walls. These results suggest the formation of screw‐type dislocation dipoles inside the dislocation cells. Such dislocation configuration can efficiently shield the lattice rotation at the given length scales of the Burgers circuit, resulting in extremely low misorientation across dislocation cells. These dislocation dipoles were reported to originate from cyclic thermal contractions and expansions during L‐PBF,^[^ ^2^, ^18^ ^]^ which facilitate the formation of well‐organized dislocation cells.

It was reported that the origin of SGBs is closely related to the solidification dendrites/cells.^[^ ^13^ ^]^ In most cases, the misorientation between solidification dendrites is very small. Nevertheless, there is a certain value of misorientation between “packets” of dendrites due to the variation of thermal gradient within melt pools.^[^ ^15^ ^]^ Consequently, additional GNDs are needed to maintain the mismatch between “packets” of solidification dendrites/cells. With the accumulation of GNDs, the cellular boundaries of “packets” would evolve into SGBs. This could explain the fact that the SGBs are always found to be overlapped with the solidification cellular boundaries. Compared with dislocation cells, higher GND densities are probed in the SGBs, and the edge‐type dislocations are dominant. Interestingly, the dislocation cells in the vicinity of SGBs show transitional dislocation types, i.e., higher GND density and larger fraction of the edge‐type dislocations compared with typical dislocation cells (additional example can be found in Figure S7, Supporting Information), which further confirms the aforementioned assumption. Nevertheless, the fraction of screw‐type GNDs varies at different SGBs. For example, only 30% of GNDs at SGB2 are screw‐typed, while a relatively high fraction (44%) of screw‐type GNDs was detected at SGB1. Furthermore, arrays of interface screw‐type GNDs with opposite signs are detected on both sides of SGB1, similar to the GND arrangements in dislocation cells (but with higher density). In contrast, the signs of both the screw‐ and edge‐type GNDs on both sides of SGB2 are the same.

It should be emphasized that the underlying mechanisms responsible for the disparity of dislocation types remain unclear. In our previous study,^[^ ^19^ ^]^ we reported the emergence of two different cellular structures within melt pools, i.e., well‐organized dislocation walls associated with Cr‐segregation (similar to the dislocation cell walls shown in Figure 4E) and loosely‐arranged dislocation structures in absence of Cr‐segregation (note that, periodic Ni‐segregation was observed, but it did not overlap with the dislocation structures). Figure 4F,G depicts quantitative results of dislocation types along these two distinct cellular structures, respectively. Interestingly, we can see that the dislocation cells associated with Cr‐segregation exhibit a predominance of screw‐type dislocations (Figure 4F), whereas the dislocation structures lacking Cr‐segregation display a prevalence of edge‐type dislocations (Figure 4G). Such experimental observations indicate a direct influence of Cr segregations on determining the dislocation types.

Note that, the enrichment of Cr was reported to reduce the stacking fault energy (SFE) in austenitic steels.^[^ ^20^ ^]^ An increase of around 1–2 at% Cr content was detected within the dislocation walls,^[^ ^17^, ^19^ ^]^ resulting in a corresponding reduction in SFE of approximately 3.5–7 mJ m^−2^, estimated by the first‐principle theory in the Fe–Ni–Cr system.^[^ ^20a^ ^]^ To reveal the impact of chemistry‐dependent SFE on dislocation stability, 3D‐DDD simulations were conducted to assess the annihilation rates of screw‐type dislocations as a function of SFE values. Note that, the specific temperature, at which the dislocation structures are created, cannot be determined. The chemical diffusion and the dislocation structure annihilation were reported to occur at temperatures exceeding 650 °C.^[^ ^13^ ^]^ Therefore, DDD simulation temperature was set at 650 °C. The SFE of 316L SS at 650 °C is estimated to be around 40 mJ m^−2^ (Figure S8A, Supporting Information). As revealed in Figure 4H, a significant improvement in the thermal stability of screw‐type dislocations is observed with even a slight decrease in SFE by ∆*γ* ≈6 mJ m^−2^ from the nominal value of 40 mJ m^−2^. This reduction of SFE mimics the case that Cr is locally enriched, as aforementioned. Consequently, during cyclic thermal contractions and expansions in the L‐PBF process, the screw‐type dislocation dipoles exhibit sustained presence in the dislocation cell walls with relatively low local SFE, owing to the Cr‐segregation. In contrast, regions lacking Cr‐enrichment witness rapid annihilation of screw‐type dislocation dipoles. Therefore, it is reasonable to deduce that the reduction in local SFE, resulting from chemical heterogeneity, plays a decisive role in the presence of screw‐type dislocations in dislocation cell walls.

### 2.5 The Crucial Role of Dislocation Types in the Thermal Stability of Cellular Structures

3D‐DDD simulations were further performed to reveal the role of dislocation types in the thermal stability of cellular structures. Based on the dislocation configurations and the residual stress distributions of SGB1 and SGB2 obtained from the experimental measurements, two different simulation boxes were established, as shown in Videos S1‐3 and S4‐6 (Supporting Information), respectively. For simplification, the initial dislocation structures of SGB1 and SGB2 were rephrased to consist of only screw‐type and edge‐type GNDs, respectively. Detailed information on the simulation setups can be found in Section 4.4.

At room temperature (RT), the dislocation configurations in both simulation boxes remain stable (Videos S1 and S4, Supporting Information), attributed to the hindrance of high friction stress at RT (Figure S8B, Supporting Information). At 600 °C, dislocation movements are observed in both simulation boxes but display quite different behaviors. Specifically, the screw‐type GNDs in SGB1 get unpinned and disperse into channels. With further simulation steps, they get trapped by dislocations with opposite signs in the channels, forming screw‐type dipoles (Video S2, Supporting Information). In contrast, cooperative movement of the edge‐type GNDs in SGB2 is observed, resulting in a sluggish migration of SGB2 (Video S5, Supporting Information). At 800 °C, the screw‐type GNDs in SGB1 show similar behavior but with higher mobility compared to that at 600 °C, ultimately getting annihilated with further simulation steps (Video S3, Supporting Information). In comparison, the cooperative movements of edge‐type GNDs in SGB2 remain prevalent at 800 °C. With further simulation steps, SGB2 is observed to migrate, in its entirety, towards to boundary (zero stress field) between tensile and compressive residual stresses. Meanwhile, the total GND density (misorientation) of SGB2 increases by collecting GNDs with same sign in the channel, as shown in Video S6 (Supporting Information). It can be seen that the simulated dislocation behaviors through 3D‐DDD (Videos S1‐6, Supporting Information) align closely with the in‐situ experimental observations displayed in Figure 3G–L, verifying the importance of dislocation types in the thermal stability of cellular structures.

**Figure 5:**
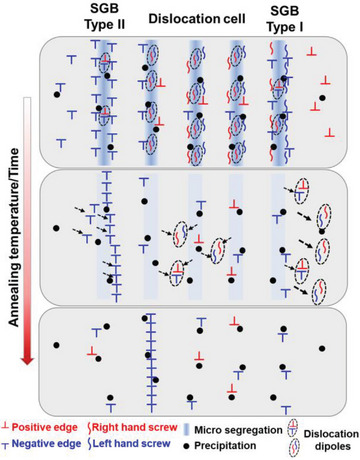
Schematics of the associated dislocation behaviors of dislocation cells and SGBs at elevated annealing temperatures. Dashed arrows suggesting the dislocation movement and the bold arrow highlighting a higher mobility.

Based on these experimental and simulation results, schematic illustrations of the dynamic evolution of three typical dislocation configurations associated with cellular structures, i.e., the dislocation cells (with screw‐type dislocation dipoles), Type I SGB (with high fraction of screw‐type GNDs, e.g., SGB1), and Type II SGB (with dominating edge‐type GNDs, e.g., SGB2), upon annealing are demonstrated in **Figure** **5**. The lower thermal stability of dislocation cell walls, compared with SGBs, can be mainly attributed to the dominance of screw‐type dislocation dipoles in the walls. Most dislocations at dislocation cell walls can be efficiently annihilated in terms of intrinsic dislocation annihilation with the assistance of cross slip.^[^ ^21^ ^]^ It is worth mentioning that the densely‐arranged screw dislocation dipoles (in the order of 10^15^ m^−2^) are extremely unstable. At RT, due to the low dislocation mobility raised by high friction stress (see Figure S8B, Supporting Information) as well as the inter‐pinning effects of solution elements,^[^ ^13^ ^]^ sessile dislocation locks,^[^ ^18^ ^]^ and oxide precipitates,^[^ ^22^ ^]^ the tendency of dislocation annihilation is strongly inhibited. With increasing annealing temperature (above 600 °C), the thermal activation leads to dislocation unpinning and, consequently, results in a fast intrinsic annihilation process in dislocation cells.

The distinct thermal stability between Type I and Type II SGBs originates from the disparity of dislocation types, as demonstrated by the simulation results in Videos S1‐3 and S4‐6 (Supporting Information). Due to the presence of high GND density in SGBs, the net signs of dislocation at SGBs are not null. Therefore, a complete annihilation of SGBs cannot be accomplished only by intrinsic dislocation annihilation. Instead, it needs the interactions between dislocations from neighboring cells, i.e., extrinsic dislocation annihilation (highlighted by dashed ellipses in Figure 5). The exhaustion rate of SGBs is, thus, strongly dependent on the unpinning ability and the mobility of dislocations. As shown in Video S1‐3 (Supporting Information), screw‐type GNDs can easily get rid of the inter‐pinning effect and disperse into channels. Consequently, the Type I SGB (with more screw‐type dislocations) shows a high migration rate. Additionally, the neighboring dislocations in the channel show opposite Burgers vectors. These factors altogether result in an effective extrinsic dislocation annihilation. In contrast, the cooperative movement of edge‐type GNDs (Video S4‐6, Supporting information) requires a much higher driving force, resulting in a low migration rate of Type II SGB.^[^ ^23^ ^]^ Moreover, since the neighboring dislocations of Type II SGB show the same sign, the misorientation increases due to the absorption of GNDs with same sign during migration. In this case, the SGBs can even evolve into LAGBs, e.g., SGB2 and SGB6 in Figure 2, leading to grain fragmentation and the relief of intra‐granular residual stress.

## 3 Conclusion

In summary, our results demonstrate a disparity of dislocation types in different dislocation structures at cellular boundaries, which imposes a pivotal effect on their thermal stability. During annealing, the screw‐type dislocation dipoles in the dislocation cell walls get efficiently exhausted via intrinsic dislocation annihilation. Moreover, for SGBs with high fraction of screw‐type GNDs, the dislocations can get rid of the inter‐pinning effect more easily, resulting in high dislocation mobility and high migration rate. In contrast, SGBs with dominating edge‐type GNDs show lower migrating rate due to the cooperative movement of edge‐type dislocations. Depending on the signs of the neighboring dislocations, the misorientation of SGBs could increase due to the accumulation of GNDs with the same signs or decrease due to the extrinsic annihilation of dislocations with opposite signs. Experimental and simulation results reveal that the dominance of screw‐type dislocations in dislocation cell walls can possibly be attributed to the reduction of local SFE, resulting from the Cr‐segregation. This crucial information sheds light on how to locally tune dislocation structures with desired dislocation types by adjusting local chemistry‐dependent SFE/heat treatment. The formation and evolution mechanisms of printed microstructure are fundamentally and technologically important, which offers a new perspective for the AM community for designing metallic materials with superior mechanical properties.

## 4 Experimental Section

### Sample Fabrication

In this study, 316L SS samples were fabricated by the L‐PBF technique in a high‐purity argon environment, via an Aixway Precision 100 machine (200 W, 25 µm beam diameter). The initial gas‐atomized 316L SS powders had a particle diameter distribution ranging from 2 to 15 µm. The dimensions of the printed samples were 55 (scan direction) × 15 (building direction) × 2 mm^3^. A multiple‐track laser scanning mode was applied with a layer thickness of 10 µm and 67° rotation between layers. After optimization of printing parameters, the sample was nearly fully dense (99.97%). The chemical composition of the printed samples was measured to be Fe‐17.02Cr‐11.02Ni‐2.13Mn‐2.36Mo‐2.72Si (in wt%). The samples for annealing treatment were cut from the as‐built plates. Before annealing, the surfaces for microstructure characterization were polished with 360–2000 grit SiC papers and 3 µm diamond suspension. High surface quality was achieved by polishing with a 50 nm colloidal suspension of SiO_2_ and followed by electrochemically polishing to remove the stress layer.

### Thermal Treatment

Annealing treatments were performed using a DIL 805A/D/T type dilatometer in a high vacuum (<10^−5^ Pa) environment. The detailed annealing history is displayed in Figure S4 (Supporting Information). Note that, to follow the microstructure evolution of the same regions, the sample underwent accumulated annealing treatments. For simplicity, here we refer to the annealing condition of the microstructure to its latest annealing step. Note that rapid heating rate (200 °C min^−1^) and short annealing time (10 min) were applied, which arise from the following considerations: the thermal stability of cellular structures was reported to be more sensitive to temperature than annealing time.^[^ ^6^ ^]^ Besides, it would be challenging to perform quasi in‐situ observation at a long annealing time since it could cause severe oxide layers on the sample surface.

### Microstructure Characterization

Both EBSD and ECCI characterizations were carried out using a Zeiss Gemini 460 scanning electron microscopy (SEM). In detail, EBSD data were collected using an Oxford Symmetry camera with a step size of 75 nm at 20 keV. ECCI was performed at an accelerating voltage of 30 keV with a semiconductor six‐quadrant backscatter electron (BSE) detector. Transmission electron microscopy (TEM) samples were prepared by focused ion beam (FIB) via FEI Helios 5 DualBeam. TEM characterizations were conducted on an FEI F200i microscope at 200 keV.

An illustration of the cECCI process is displayed in Figure S1 (Supporting Information). To intuitively elucidate the relationship between local orientation and the corresponding channeling contrast, a simulated electron channeling pattern (ECP) of the selected grain was constructed by the TOCA software, as shown in Figure S1B (Supporting Information). After stage calibration, the diffraction conditions can be predicted based on the orientation information obtained from the EBSD measurement. Detailed information on the calibration process can be found elsewhere.^[^ ^14^, ^24^ ^]^ By this approach, the diffraction conditions of different local regions (numbered A to E in Figure S1A, Supporting Information) can be obtained, which are marked by crosses with corresponding colors on the simulated ECP. We can see that the predicted diffraction conditions fit quite well with the real channeling contrasts. For example, the diffraction conditions of regions A and C satisfy the so‐called “two‐beam” conditions (crosses coincident with the Kikuchi band lines). Consequently, regions A and C reach the optimum contrast, and the dislocation arrangements within cellular structures can be resolved, as displayed in Figure S1C,D (Supporting Information), respectively. In contrast, in regions B and D, dislocations can be hardly visualized due to the strong backscattering diffraction conditions (crosses lying inside Kikuchi bands).

HR‐EBSD (angular resolution of 0.005°)^[^ ^14e^ ^]^ was carried on to measure the local misorientation and GND distribution of cellular structures. To acquire high‐quality EBSD patterns (EBSPs), long exposure time (10 ms), low gain (1), and 10 frames averaging were applied. The reference pattern was selected from the point with the lowest kernel average misorientation (KAM) value. Cross‐correlation processing of EBSPs was performed using commercial Crosscourt 4 software to measure the elastic strain and lattice rotation.^[^ ^14b^ ^]^ This analysis enables the measurement of misorientation angle in the order of 0.005° or even higher.^[^ ^14e^ ^]^ Note that there are significant challenges in measuring the elastic strains/residual stress of L‐PBF samples, which is inherently related to the large lattice rotation arising from the high residual stress.^[^ ^8^ ^]^ To overcome this issue, we subdivided the grain into several regions with a reference tolerance angle of 2.5° and selected individual reference patterns for each region (highlighted as black dots in Figure 1D). Furthermore, remapping algorithm was conducted to reduce the artefacts caused by large lattice rotation in measuring the small elastic strains.^[^ ^14c^ ^]^ The GND density can be calculated by using the Crosscourt 4 based on the lattice rotation and elastic strain gradients.^[^ ^25^ ^]^ It is worth mentioning that there are 18 GND types (12 edge and 6 screw dislocations) in the studied FCC material while only six curvature tensors can be acquired by the 2D EBSD measurement (missing out‐of‐plane components). As a result, the solution of GND types is not unique. To solve this issue, the algorithm applied in Crosscourt 4 is to seek the combination of GND arrangements that satisfies the obtained strain tensor with the minimum total GND line energy.

### Discrete Dislocation Dynamics Simulation Setup

3D‐DDD simulations were performed using the open‐source code ParaDiS.^[^ ^26^ ^]^ Within the ParaDiS framework, dislocations are discretized into a series of interconnected dislocation segments. The velocity of dislocation segments, *v*, depends on the resolved shear stress, *τ*, and the lattice friction stress, τ_*f*_, which can be expressed as:^[^ ^27^ ^]^

(1) $$v=\left\{\begin{array}{cc} \frac{b\left(\tau -\tau_{f}\right)}{B}, & \tau >\tau_{f}\\ 0, & else \end{array}\right.$$

where *b* is the magnitude of the Burgers vector, and *B* = 10^−4^ Pa s is the drag coefficient.^[^ ^28^ ^]^ The material parameters of 316L SS were determined based on the literature.^[^ ^2^, ^29^ ^]^

To accurately model the dislocation structure evolution in 316L SS upon annealing, DDD simulation framework incorporates two temperature‐dependent factors, i.e., the dislocation cross‐slip probability *P* and the lattice friction stress τ_f_. The cross‐slip probability *P* of the local dislocation segment can be calculated through an Arrhenius‐like relationship:^[^ ^30^ ^]^

(2) $$P=1-\exp\left[-\omega m\frac{L}{L_{0}}\exp\left(-\frac{\Delta E}{kT}\right)\Delta t\right]$$

where *ω* = 10^13^ s^−1^ represents the attempt frequency of dislocations to overcome the barriers, *m* = 10^6^ is a scaling factor that considers the differences in experimental and DDD simulation scales, *L* denotes the length of the local dislocation segment, *L*_0_ = 1000 nm is the reference dislocation length, *ΔE* is the cross‐slip activation energy, *k* = 1.38 × 10^−23^ J K^−1^ is the Boltzmann constant, *T* is the temperature, and Δ*t* = 2.5 × 10^−12^ s is the simulation time step. Note that, the *ΔE* values for 316L SS at different temperatures are currently unavailable in the existing literature. In the model, *ΔE* for 316L SS is derived similar to that for nickel,^[^ ^31^ ^]^ following the relationship *ΔE* ∝ (*Gb*/16*πγ*)[ln(*Gb*/16*πγ*)]^0.5^ specific to FCC materials.^[^ ^32^ ^]^ Here, the SFEs, *γ*, of 316L SS at varying temperatures, are determined by linearly fitting the critical twinning stress and first‐principle calculation results,^[^ ^33^ ^]^ as depicted in Figure S8A (Supporting Information). Based on these parameters mentioned above, the variation of cross‐slip probability with increasing temperature can be determined. Furthermore, the lattice friction stress τ_f_ at elevated temperatures is obtained by fitting the experimental results with an exponential equation,^[^ ^34^ ^]^ as displayed in Figure S8B (Supporting Information). As the simulation is proceeded, *P* and τ_f_ with varying temperatures are determined based on these fitting results.

All the simulation boxes are cubic with a length of 1200 nm (4650 *b*), the periodic boundary condition was applied in three directions, and 20 000 simulation steps were performed. To explore the underlying mechanism for the dominance of screw‐type dislocations in the dislocation cell walls, the annihilation rates of screw‐type dislocations with SFE ranging from 30 to 60 mJ m^−2^ using 3D‐DDD simulations were examined. The temperature is set as 650 °C, beyond which chemical diffusion is considered to be activated.^[^ ^13^ ^]^ The initial configuration of dislocation wall was comprised of pure screw‐type dislocations with a total density of 10^15^ m^−2^ (here the dislocation density is defined as the total dislocation length divided by the volume of the dislocation cell wall) and half of the dislocations are with opposite Burgers vectors. Five initial configurations of dislocation wall are used to ensure statistically representative results.

To reveal the role of dislocation types on the thermal stability of dislocation structures, 3D‐DDD simulations were conducted to show the dynamic evolution of dislocation structures with different dislocation types (screw or edge, the sign of Burgers vectors, etc.) and at different temperatures (RT, 600 °C, and 800 °C). Dislocation configurations and the distribution of residual stress of the simulation models were established according to the experimental measurements. For simplification, the initial dislocation structure of SGB1 was set to be composed of pure screw‐type dislocations with a density of 6.95 × 10^13^ m^−2^ in the simulation model. The simulation box contains four dislocation cell walls, each with a wall thickness of 30 nm and spacing of 300 nm. Additionally, an equal number of dislocations with opposite signs were distributed in the channel regions. Note that, owing to the so‐called “reference pattern problem” and the large lattice rotation, the residual stress measured by HR‐EBSD exceeds the yield stress of the studied materials. To assure the rationality of our simulation process, we proportionally scale down the measured residual stress to fall below yield stress. In detail, σ_11_ with value of ‐150 MPa (compressive) was homogeneously distributed across SGB 1. For SGB 2, initial dislocation configuration of pure edge‐type dislocations with density of 4.86 × 10^13^ m^−2^ was established in the simulation box. Experimental measurements reveal that the dislocations of SGB 2 and its neighbors have the same sign of Burgers vectors, and only GB migration is observed (see Figure 3G–L). For simplification, only one SGB wall was constructed in the simulation box. Moreover, residual stress maps of σ_11_ and σ_22_ (Figure S6A,B, Supporting Information) reveal apparent values near SGB 2, and both vary significantly across SGB 2. Accordingly, both σ_11_ and σ_22_ were considered in the simulation box, which are set in the upper half and bottom half of the simulation box, respectively. The detailed set‐up of residual stress is demonstrated in Equation (3):

(3) $$\sigma_{e}=\left\{\begin{array}{cc} -150\left[\begin{array}{ccc} 0.028 & -0.157 & 0.048\\ -0.157 & 0.889 & -0.272\\ 0.048 & -0.272 & 0.083 \end{array}\right], & \begin{array}{c} \mathrm{upper}\;\mathrm{half}\;\mathrm{of}\;\mathrm{the}\\ \;\mathrm{simulation}\;\mathrm{box} \end{array}\\ -300\left[\begin{array}{ccc} 0.222 & 0.157 & 0.385\\ 0.157 & 0.111 & 0.272\\ 0.385 & 0.272 & 0.667 \end{array}\right], & \begin{array}{c} \mathrm{bottom}\;\mathrm{half}\;\mathrm{of}\;\mathrm{the}\\ \;\mathrm{simulation}\;\mathrm{box} \end{array} \end{array}\right.$$

where the matrix is a measure of the relative orientation of the DDD simulation box with respect to the experimental sample coordinate system where the residual stress component is measured.

## Conflict of Interest

The authors declare no conflict of interest.

## Author Contributions

Conceptualization: D.A., Y.X., Y.M.; Methodology: D.A., Y.X., J.Y., X.Z., S.Z.; Investigation: D.A., Y.X., Z.L., Y.M., R.L., X.L., J.C.; Visualization: D.A.; Supervision: D.A., Y.M., X.L., J.C., S.Z.; Writing—original draft: D.A.; Writing—review & editing: D.A., J.Y., X.Z., Y.M., S.Z.; Founding acquisition: D.A., X.Z., R.L., J.C.

## Supporting information

Supporting Information

Supplemental Video 1

Supplemental Video 2

Supplemental Video 3

Supplemental Video 4

Supplemental Video 5

Supplemental Video 6

## Data Availability

The data that support the findings of this study are available from the corresponding author upon reasonable request.
