# Supplementary material for: The Role of Dislocation Type in the Thermal Stability of Cellular Structures in Additively Manufactured Austenitic Stainless Steel
Source: Adv Sci (Weinh). 2024 Jul 1;11(33):2402962. doi: 10.1002/advs.202402962 (PMC11434014; doi:10.1002/advs.202402962)
Supplement: Supplementary file 1 — Supporting Information [file ADVS-11-2402962-s007.pdf]

ADVANCED SCIENCE
Open Access

Supporting Information

for *Adv. Sci.*, DOI 10.1002/advs.202402962

The Role of Dislocation Type in the Thermal Stability of Cellular Structures in Additively Manufactured Austenitic Stainless Steel

Dayong An, Yao Xiao, Junshi Yu, Xu Zhang*, Zan Li, Yan Ma*, Rui Li, Xianhong Han, Xifeng Li, Jun Chen and Stefan Zaefferer*

Supporting Information

The role of dislocation type in the thermal stability of cellular structures in additively manufactured austenitic stainless steel

Dayong An, Yao Xiao, Junshi Yu, Xu Zhang*, Zan Li, Yan Ma*, Rui Li, Xianhong Han, Xifeng Li, Jun Chen, Stefan Zaefferer*

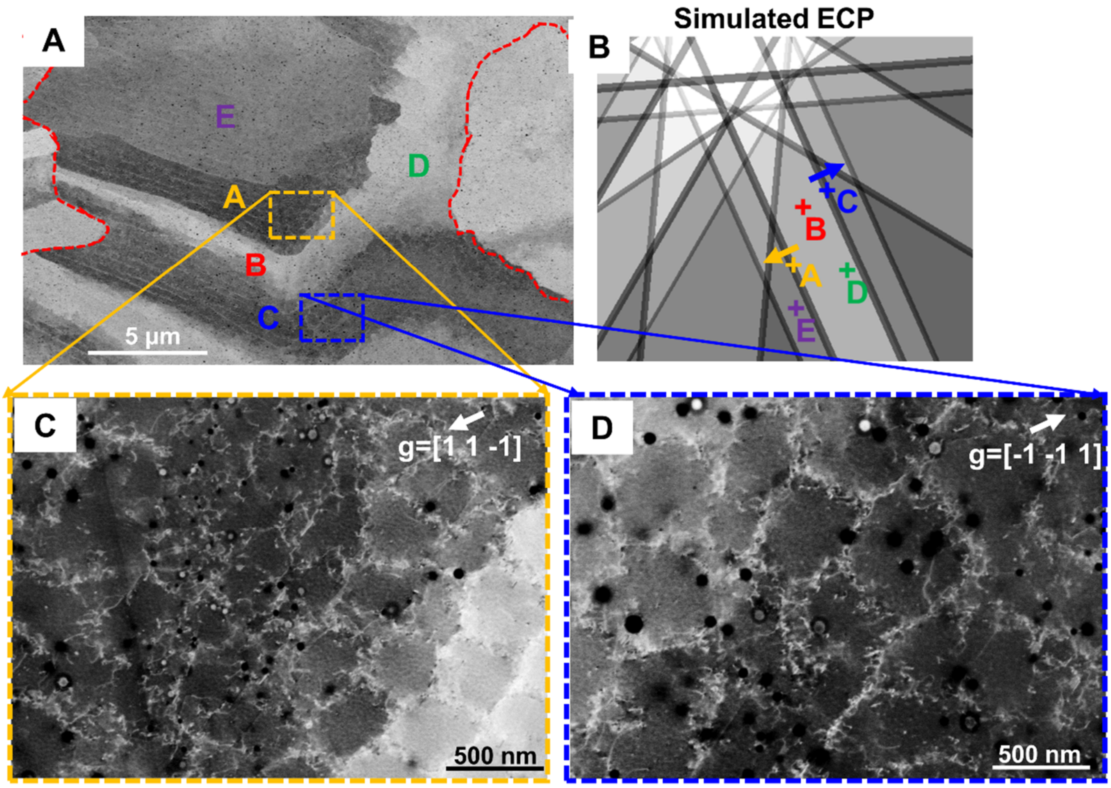

Figure S1 Illustration of ECCI under controlled diffraction conditions (cECCI). (A) ECC image of the selected grain. The regions with different channeling contrasts are labeled A to E, correspondingly. (B) A simulated ECP with orientation near the selected grain. After stage calibration, the specific diffraction conditions of regions A to E are marked by crosses with corresponding colors on the simulated ECP map. (C) and (D) Enlarged ECC images of the regions selected in Region A and C, respectively. The white arrows indicate the direction of **g** vectors of regions A and C.

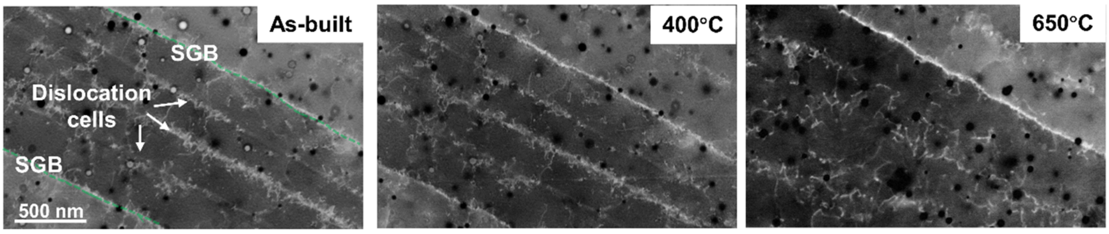

Figure S2 Dislocation-scaled microstructure evolution at elevated annealing temperatures captured by ECCI.

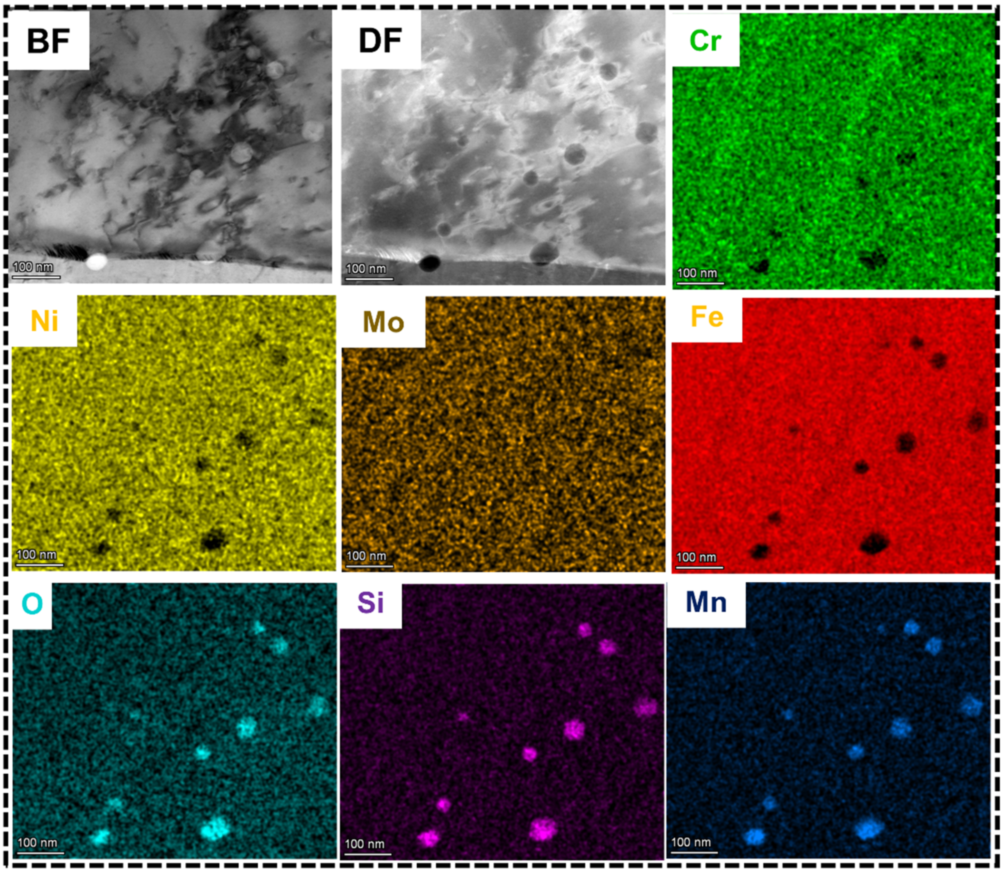

Figure S3 Scaning transmission electron microscope (STEM)-energy dispersive spectroscopy (EDS) maps of the cellular structure.

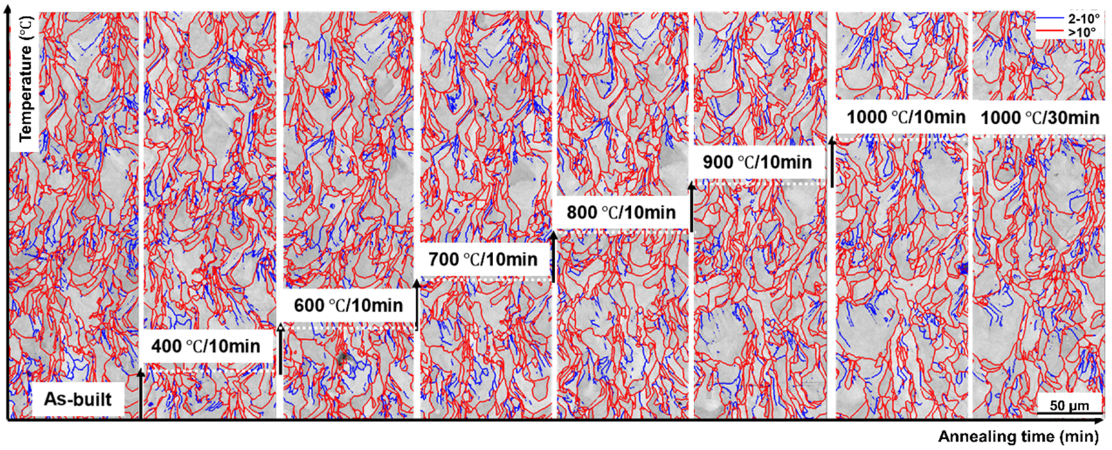

Figure S4 Overview of microstructure evolution at elevated annealing temperatures. The annealing history is displayed on the corresponding maps.

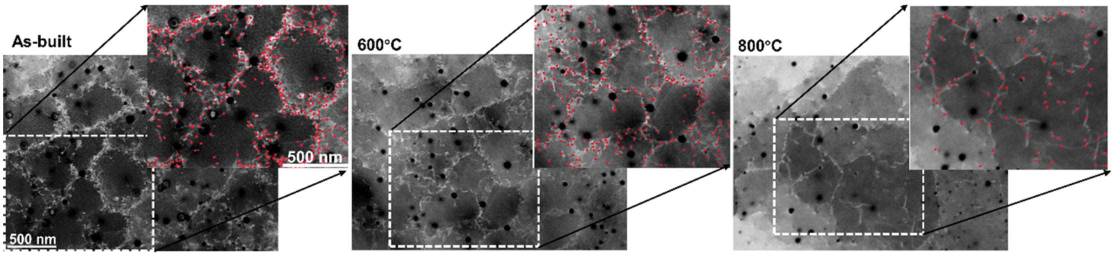

Figure S5 Illustration of dislocation density estimation process. The average dislocation density of cellular structure, including the cellular boundaries and interiors, is calculated by counting the intersections of individual dislocations with surface (red dots) and then dividing the total number by the measured area.

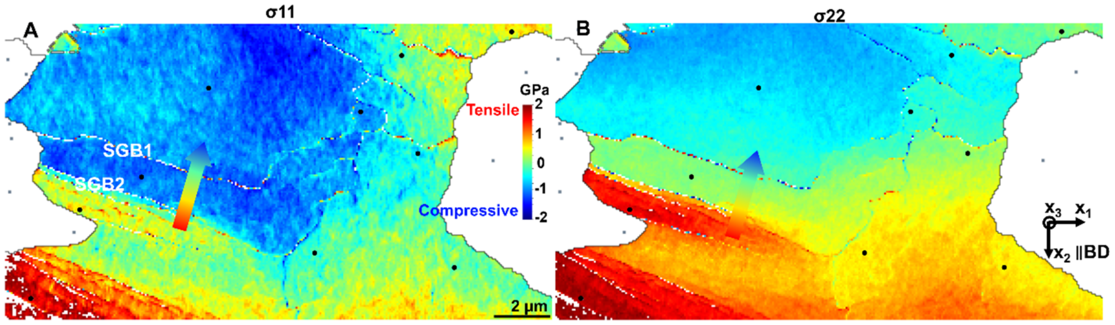

Figure S6 Maps of the primary residual stress for the grain showing in Figure 1 (B). The red and blue colors demonstrating the tensile and compressive stresses, respectively.

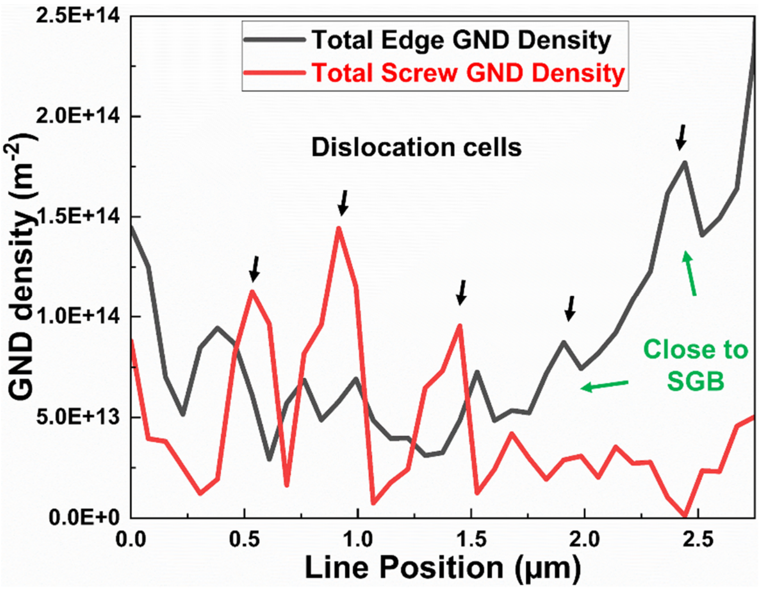

Figure S7 Profiles of the total edge and total screw GND densities along line2 in Figure 1(B2).

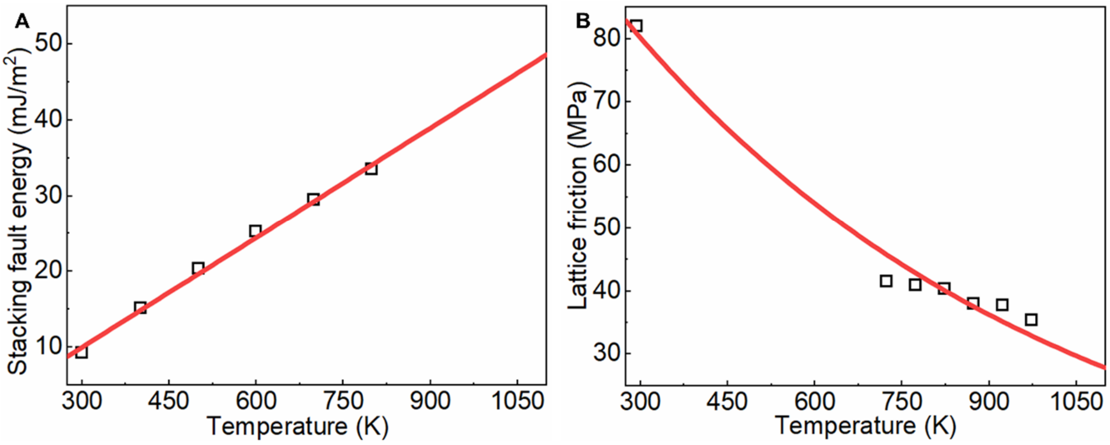

Figure S8 (A) Linear fitting of the SFE of 316L SS versus temperature.[33] (B) Exponential fitting of the lattice friction of 316L SS versus temperature.[34]
